# Integrating Global Citizen Science Platforms to Enable Next-Generation Surveillance of Invasive and Vector Mosquitoes

**DOI:** 10.3390/insects13080675

**Published:** 2022-07-27

**Authors:** Ryan M. Carney, Connor Mapes, Russanne D. Low, Alex Long, Anne Bowser, David Durieux, Karlene Rivera, Berj Dekramanjian, Frederic Bartumeus, Daniel Guerrero, Carrie E. Seltzer, Farhat Azam, Sriram Chellappan, John R. B. Palmer

**Affiliations:** 1 Department of Integrative Biology, University of South Florida (USF), Tampa, FL 33620, USA; connormapes@usf.edu (C.M.); daviddurieux@usf.edu (D.D.); kjrivera@usf.edu (K.R.); 2 Woodrow Wilson International Center for Scholars, Washington, DC 20007, USA; alex.long@wilsoncenter.org (A.L.); anne.bowser@gmail.com (A.B.); 3 Institute for Global Environmental Strategies, Arlington, VA 22202, USA; rusty_low@strategies.org; 4 Department of Political and Social Sciences, Universitat Pompeu Fabra, 08005 Barcelona, Spain; berj.dekramanjian@upf.edu (B.D.); john.palmer@upf.edu (J.R.B.P.); 5 Centre d’Estudis Avançats de Blanes (CEAB-CSIC), 17300 Blanes, Spain; fbartu@ceab.csic.es; 6 Centre de Recerca Ecològica i Aplicacions Forestals (CREAF), 08193 Cerdanyola del Vallès, Spain; 7 Institució Catalana de Recerca i Estudis Avançats (ICREA), 08010 Barcelona, Spain; 8 iNaturalist, California Academy of Sciences, San Francisco, CA 94118, USA; carrie@inaturalist.org; 9 Department of Computer Science and Engineering, University of South Florida, Tampa, FL 33620, USA; farhatbinte@usf.edu (F.A.); sriramc@usf.edu (S.C.)

**Keywords:** artificial intelligence, citizen science, computer vision, geographic information systems, invasive species, machine learning, mosquito monitoring, smartphone, vector-borne disease, vector surveillance

## Abstract

**Simple Summary:**

The mosquito is the deadliest animal on earth, transmitting diseases that cause nearly a million deaths and >700 million infections each year. Yet only relatively few mosquito species are “vectors” that transmit diseases. Unfortunately, identifying these vector species is a difficult and time-consuming manual process. A promising and scalable solution involves “citizen science”, whereby the general public provides images of mosquito specimens and breeding habitats using smartphones. However, data from such previous efforts have lacked the necessary integration for a thorough and global understanding of mosquito presence. Here, we standardize and combine data from multiple international citizen science apps—Mosquito Alert, iNaturalist, and GLOBE Observer’s Mosquito Habitat Mapper and Land Cover—to aid researchers, mosquito control personnel, and policymakers. We also launched coordinated media campaigns that generated unprecedented numbers and types of observations, including successfully capturing the first images of targeted invasive and vector species. Additionally, we used citizen science imagery to develop artificial intelligence software to automatically identify the species and anatomical regions of mosquitoes. Ultimately, we establish a new surveillance system to serve as a united front to combat the ongoing threat of mosquito-borne diseases worldwide.

**Abstract:**

Mosquito-borne diseases continue to ravage humankind with >700 million infections and nearly one million deaths every year. Yet only a small percentage of the >3500 mosquito species transmit diseases, necessitating both extensive surveillance and precise identification. Unfortunately, such efforts are costly, time-consuming, and require entomological expertise. As envisioned by the Global Mosquito Alert Consortium, citizen science can provide a scalable solution. However, disparate data standards across existing platforms have thus far precluded truly global integration. Here, utilizing Open Geospatial Consortium standards, we harmonized four data streams from three established mobile apps—Mosquito Alert, iNaturalist, and GLOBE Observer’s Mosquito Habitat Mapper and Land Cover—to facilitate interoperability and utility for researchers, mosquito control personnel, and policymakers. We also launched coordinated media campaigns that generated unprecedented numbers and types of observations, including successfully capturing the first images of targeted invasive and vector species. Additionally, we leveraged pooled image data to develop a toolset of artificial intelligence algorithms for future deployment in taxonomic and anatomical identification. Ultimately, by harnessing the combined powers of citizen science and artificial intelligence, we establish a next-generation surveillance framework to serve as a united front to combat the ongoing threat of mosquito-borne diseases worldwide.

## 1. Introduction

Citizen science, also known as community science, brings the public into the process of scientific research through participation in data collection. A complex and often-contested term [1,2], citizen science is conducted by a range of stakeholders with different scientific, environmental, and/or social goals, including advancing research, creating a more informed and engaged society, and impacting public policy or other decision-making. Today, there are thousands of citizen science projects active around the world (e.g., scistarter.org, citizenscience.gov, both accessed on 30 June 2022), from those led by local community groups to those led by major research institutions working across multiple continents. Many citizen science studies are aimed at monitoring insects, and include invasive species as well as data validation (e.g., [3,4,5,6,7]).

The potential contribution of citizen science to address health threats has attracted the attention of scientists and public health professionals [8,9,10]. This is especially the case when it comes to the role that citizen scientists can play in reducing the substantial global burden of mosquito-borne diseases [11]. Mosquitoes are the deadliest animals on the planet, long responsible for substantial morbidity and mortality to humans and many other animals. Indeed, over half the human population is at risk for mosquito-borne diseases, which are responsible each year for >700 million infections and nearly one million deaths [12,13]. Given the general lack of vaccines and cures, adequate mosquito control is a critical community defense against these pathogens, and there is a nearly universal need for increased and sustained mosquito surveillance and habitat mitigation worldwide.

Broad access to mobile devices has enabled the activation of citizen scientists as a scalable and cost-effective solution [14] to vastly improve the spatial and temporal coverage of mosquito observations. Such comprehensive surveillance is particularly critical for monitoring the range and expansion of invasive vectors [14]. For combatting mosquito-borne diseases, the volume and velocity [15] of surveillance data available through crowdsourcing efforts can be of substantial value to mosquito control and public health professionals. Engaging the public in routine mosquito surveillance in and around their homes and communities has the potential to provide data at a frequency and geographic resolution otherwise impossible because of cost and other constraints [16]—including data on private property, which can be useful to municipal mosquito control agencies from an operational perspective.

Beyond the collection of valuable mosquito data, public engagement can also promote helpful behaviors to manage health crises. For instance, projects can ask participants to report when they remove standing water, which serves as a potential habitat for mosquito breeding (i.e., oviposition, or egg laying) as well as the larvae and pupae [17]. Citizen scientists also share their experiences and knowledge with their communities, improving local awareness about protective measures that reduce risks from mosquito-borne diseases [18]. These actions, in turn, stimulate community involvement in vector control [11,19] while simultaneously empowering individuals to be agents of change—promoting environmental justice and reducing health disparities between communities. This is especially true for rural and remote communities where no active mosquito monitoring programs exist [20].

In the last decade, more than a dozen citizen science mosquito surveillance projects have been established that collect, report, and analyze data. The unique characteristics of each of these citizen science projects, when examined together, provide an impressive international field experiment that explores the effectiveness of different community engagement strategies, app interfaces, data requests, and tools. The study herein focuses on three mobile apps that are used globally: Mosquito Alert (mosquitoalert.com, accessed on 30 June 2022), GLOBE Observer (observer.globe.gov, accessed on 30 June 2022), and iNaturalist (inaturalist.org, accessed on 30 June 2022). Other mosquito projects are restricted to a single country or region, for example: Argentina (Caza Mosquitos [21]), Australia (Mozzie Monitors [22]), France (iMoustique^®^ [23]), Germany (Mosquito Mapper [24] and Mückenatlas [25,26,27]), Italy (ZanzaMapp [28]), the Netherlands (Muggenradar [23]), Portugal (MosquitoWEB [23]), Rwanda [20], Spain (AtrapaelTigre.com [23]), Tanzania [29], the UK (Mosquito Reporting Scheme and Mosquito Watch [23]), and the USA (Mosquito Stoppers [30], Great Arizona Mosquito Hunt [31], North American Mosquito Project [32], and Kidenga [33]).

There are often advantages to be gained from reusing such citizen science data and combining them across monitoring activities, research projects, and/or geographic regions. However, like with many new and open innovations, the standardization of common best practices is difficult to implement. Many citizen science projects prioritize collecting and analyzing data that are tailored for a particular purpose, rather than prioritizing collecting, analyzing, and sharing data for eventual reuse. The former situation leads to inconsistent data standards and structures, and to technical obstacles to obtaining, merging, and analyzing [34] disparate datasets. Such challenges drove the inception of the Global Mosquito Alert Consortium [11], which seeks to connect those in the international citizen science community through common protocols involving real-time monitoring of breeding habitats, mosquitoes, and bites, along with mosquito biodiversity approaches—all of which are put into place by a bundle of apps that can be customized to a specific locality via language options.

### Objectives

With that background in mind, we sought to leverage work conducted on citizen science mosquito monitoring, and pursue three main objectives:

I. **Integrate datasets from different citizen science projects into a single, interoperable dashboard** to support current and future global surveillance of invasive and vector mosquitoes. By harmonizing (i.e., standardizing and combining) multiple updating data streams into an easily accessible and free online format, we predict that these efforts will add value by contributing to monitoring and investigative outcomes beyond what a single platform might achieve. Benefits will arise from reusing and pooling information across disparate spatial and temporal scales as well as across a broader set of observations, from land cover and breeding habitats to larvae, adults, and bites.
II. **Target four invasive and vector species of *Aedes* (*Ae.*) mosquitoes** for observation through a multi-platform promotional campaign and direct recruitment of citizen scientists. Prior work suggests that citizen scientist observations of mosquitoes are reliable measures of relative abundance and human–mosquito interactions [14,30]. Thus, we also aim to generate and reuse species presence data to populate a national risk model for *Ae. albopictus* (Spain) and to validate a local habitat model for *Ae. aegypti* (Tampa Bay area, FL, USA). These two species are the primary vectors of dengue, Zika, yellow fever, and chikungunya [13]. *Ae. scapularis* [35] and *Ae. vittatus* [36] were recently detected via traps in south Florida and the Caribbean, respectively. Both invasive species have been identified as vectors [36,37] and present new challenges to local vector control organizations for the surveillance and containment of these exotics. Therefore, we also aim to generate new citizen science observations for these two species for both general surveillance and early warning purposes, especially considering that there are no iNaturalist observations of *Ae. scapularis* in the USA or *Ae. vittatus* worldwide. We predict that our campaigns will yield at least one observation of an invasive *Aedes* in a new area for a respective platform, and that the citizen science data will serve as a useful complement to trap-based data for the risk and habitat models.
III. **Reuse citizen science images to train and test artificial intelligence (AI) algorithms that identify mosquito species.** These efforts will build on our prior image recognition work, which has demonstrated that smartphone photos of mosquitoes can be accurately classified [38,39,40]. We aim to integrate images across multiple platforms to create training datasets that are balanced across classes. While photos of all mosquito species are beneficial, the need is especially critical for the aforementioned *Ae. scapularis* and *Ae. vittatus*. We predict that these efforts will serve to catalyze a longer-term objective of deploying real-time AI in future surveillance efforts.

## 2. Materials and Methods

### 2.1. Mosquito Alert

Mosquito Alert is a citizen science system that has been initiated and managed by public research institutions, with the goal of bringing the general public together with academics, public health authorities, and other stakeholders to study and control the spread of disease-spreading mosquitoes worldwide [14,16,41,42]. The project was launched in June 2014 with an initial focus on *Ae. albopictus* in Spain [14,16]. It was expanded in 2016 to include *Ae. aegypti*, and again in 2020 to include *Ae. japonicus*, *Ae. koreicus*, and *Culex* spp.

**Figure 1:**
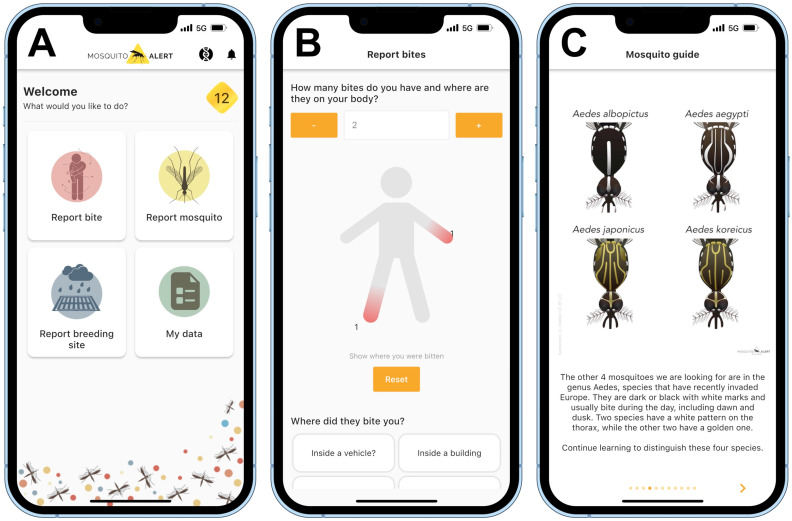
Mosquito Alert user interface. Screenshots illustrating the mobile app’s dashboard (**A**), bite reporting module (**B**), and species identification guide (**C**).

Citizen scientists connect to the system through an app that is available for iOS and Android devices, has an interface in 18 languages, and can be used anywhere in the world. The in-app interface and species identification guide are shown in Figure 1. Participants provide information from their smartphones by sending reports about adult mosquitoes and mosquito breeding habitats (aka. sites) that they encounter. All adult mosquito reports accompanied by photos are validated via a web-based interface by a team of entomologists, who annotate each report with their assessment of which (if any) of the targeted species is shown in the photos. Since 2020, participants have also been able to report biting incidents.

Mosquito Alert has received more than 82,128 reports from 33,091 participants in 168 countries to date, as calculated in the automated participation report at github.com/Mosquito-Alert/participation/blob/8e1658914e4aae3d4f0cca6a22d79a6dccd1b199/index.html (accessed on 21 April 2022). The latest participation report (updated daily) is available at mosquito-alert.github.io/participation/ (accessed on 21 April 2022). In 22 of those countries, the app has been used for formal vector surveillance through a network created and supported by the Aedes Invasive Mosquito Cooperation In Science & Technology (AIM-COST) Action and Versatile Emerging Infectious Disease Observatory (VEO) projects funded by the European Commission. One strength of the approach that Mosquito Alert takes is its ability to provide mosquito surveillance and population estimates over large geographic areas at a relatively low cost compared to traditional methods [14]. Mosquito Alert’s role in providing early warning of new areas invaded by targeted species has been well-documented in the case of *Ae. albopictus* [14], as well as the first detection of *Ae. japonicus* in Spain [41,42] and *Ae. vittatus* in Galicia [43], and this list continues to grow.

### 2.2. GLOBE Observer

The Global Learning and Observations to Benefit the Environment (GLOBE) Program has been providing students and the public worldwide with the opportunity to meaningfully contribute to our understanding of the Earth system and global environment since 1995 (globe.gov, accessed on 30 June 2022). The GLOBE Program’s GLOBE Observer smartphone app was launched in 2016 to facilitate field-based data collection and broaden the reach of NASA science to the general public [44]. The app is currently available in 14 languages and has been used in 86 countries. Citizen scientists using the GLOBE Observer app provide in-situ ground reference data needed by scientists to analyze and interpret digital data derived by sensors on satellite platforms.

**Figure 2:**
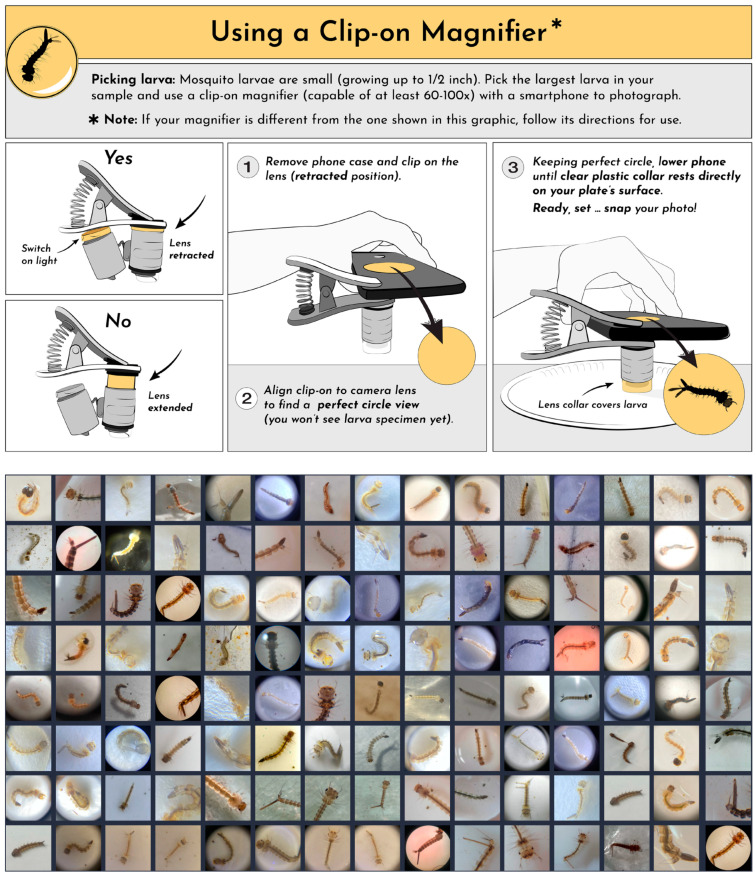
Images from the Mosquito Habitat Mapper’s 2021 Mosquito Habitat Photo Challenge. (**Top**) Panel from the instructional materials for citizen scientists, demonstrating the proper way to use an inexpensive 60× clip-on lens with a smartphone to photograph mosquito larvae. The full two-page PDF is included as File S1. (**Bottom**) A selection of citizen science images submitted through the Mosquito Habitat Mapper app during the challenge.

The app currently includes four tools on its platform, including the Mosquito Habitat Mapper and the Land Cover tool. In response to the Zika epidemic in Brazil in 2016, the Mosquito Habitat Mapper was released in 2017 to support community engagement with the identification and elimination of three medically important genera (*Aedes*, *Anopheles*, and *Culex*) and two species in particular (*Ae. albopictus* and *Ae. aegypti*) [17]. The Mosquito Habitat Mapper enables users to geolocate and photograph standing water habitats and mosquito larvae, as well as document counts of larvae and pupae and the presence/absence of eggs and nearby adults [45]. To properly photograph a larva, the citizen scientist needs to use an inexpensive (USD 5–10) clip-on macro lens with at least 60x magnification (Figure 2) or, alternatively, use a microscope.

Larval specimens can then be identified as one of the three aforementioned genera using a built-in dichotomous pictorial key, which presents the field photos alongside diagrams and photos for comparison. While most citizen scientists submitting larval images do not attempt to identify the genus, this feature has encouraged users to look carefully at their specimens, with the result that the incidence of macroinvertebrates misidentified as mosquitoes is very low—well below one percent, based on a recent expert photo validation of container mosquitoes from four countries in Africa [45]. Taxonomic identifications by citizen scientists are reported in the Mosquito Habitat Mapper database but require expert validation. Scientists who are interested in such identifications are supplied with voucher photos for each specimen to screen for accuracy.

All photos are screened prior to inclusion in the database. This process is conducted manually by members of the project team and excludes all photos that do not meet the requirements that all accepted photos are either mosquito larvae or habitats. In addition, any photos that include human faces, advertising signs, or license plates, for example, are rejected during photo validation. The larval photos are ported into the database at the resolution at which they were captured. Curated datasets are posted on our website, geospatial.strategies.org (beta, accessed on 30 June 2022), enabling others to access data that has been validated by experts.

After these data are recorded, users are asked to report if they can mitigate the mosquito habitat by removing, dumping out, or covering the water-filled container they encountered. This action, known as source reduction, has a measurable impact on the transmission of mosquito-borne diseases [46,47,48] and enables the citizen scientist to not only contribute to science but also to tangibly reduce the risk of disease in their community.

The Land Cover tool makes it convenient for the citizen scientist to report the ecological conditions immediately surrounding the standing water habitat. A built-in compass guides the user in collecting six voucher photos centered on the habitat location (north, south, east, west, up, down). If desired, the user can describe the land cover in each direction by assigning a percent value to observed land cover features. These data autogenerate a land cover class aligned with the Modified UNESCO Classification [49]. A recent update to the GLOBE Observer app now encourages citizen scientists to collect coincident Mosquito Habitat Mapper and Land Cover data, which provides utility for future analyses.

### 2.3. iNaturalist

iNaturalist is a social network that connects people to nature through sharing observations of biodiversity and crowdsourcing identifications. The platform is accessible worldwide and translated into more than 40 languages. Since the launch of iNaturalist on 20 March 2008, nearly 3 million people have contributed more than 121 million observations representing ~400,000 species across all taxonomic groups. About 25% of observations are insects, and, at writing, there are ~66,000 observations of mosquitoes identified to 386 species and covering all inhabited continents. Data from iNaturalist are used extensively in publications on ecology, conservation, climate change, evolution, species distributions, biogeography, and agriculture. Due to its geographic and taxonomic diversity, iNaturalist is the most-cited dataset among the tens of thousands of datasets tracked by the Global Biodiversity Information Facility (GBIF). iNaturalist data are frequently used to track the spread of invasive species, both academically in publications and by land managers for immediate action (e.g., [50,51,52]). Observations are separated into “Casual” and “Verifiable”, with the latter requiring a date, coordinates, and an image or audio recording of a naturally occurring (i.e., wild) organism. Observations are classified as “Research Grade” if they have agreement of more than ⅔ of the identifications from community members at the species level, or if the observation is at a taxonomic level finer than family and the community has voted that a finer identification is not possible. A Research Grade observation must have at minimum two identifications (e.g., from the observer and one other member of the community).

In the case of identification disagreements, the most specific shared classification is used for the observation. For example, if someone identifies an observation as *Ae. albopictus* and another person identifies it as *Ae. aegypti*, the identification will remain at *Aedes* until more people weigh in and the ⅔ agreement threshold is crossed for a species. Similarly, if someone submits a photo of a caterpillar with the identification of family Culicidae, an improved identification of order Lepidoptera would bring the identification to subclass Pterygota, which is the shared subclass of Culicidae and Lepidoptera in the iNaturalist taxonomy. In this way, identifications can be corrected and refined through the community identification process.

iNaturalist maintains a website, mobile apps for iOS and Android, and an application programming interface (API) that can be used to build other apps for data retrieval or submission. The platform also provides the ability to subscribe to a particular taxon, place, and user and receive alerts of any new observations. iNaturalist regularly prepares snapshots of a subset of data for GBIF [53], the Amazon Web Services Open Data Program, and other data partners. The tens of millions of identified photos have been used to train an AI model to suggest identifications for over 38,000 taxa [54]. Externally, the voluminous and Research Grade observations have been used as training data for AI models that identify insects such as bees [55] and bee mimics [56], but there are no known published studies on mosquito identification using iNaturalist data.

### 2.4. Campaigns

**Figure 3:**
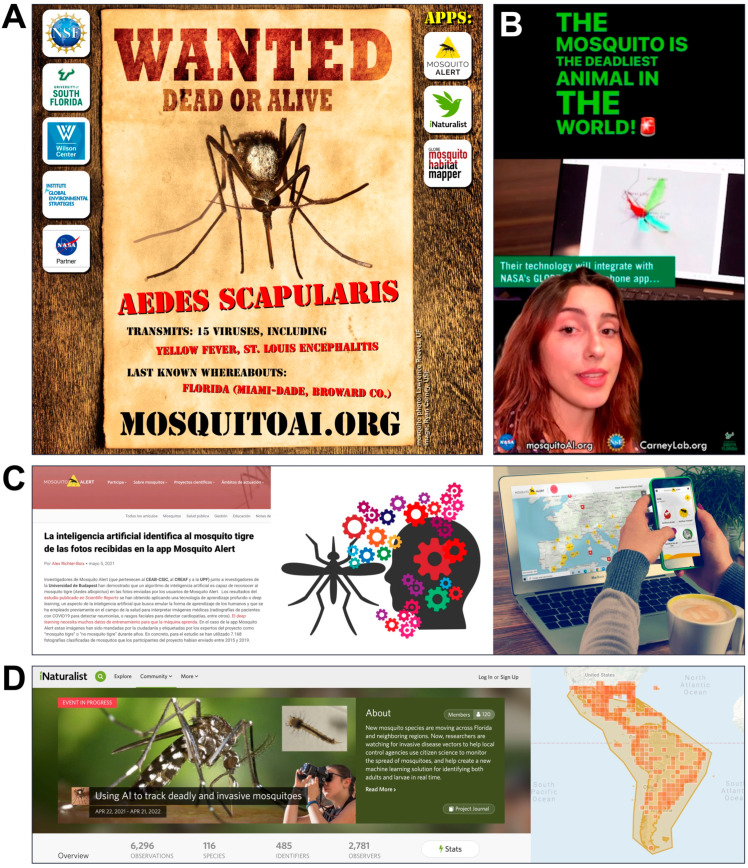
Outreach materials from our citizen science campaigns. (**A**) “Wanted” poster of *Aedes scapularis*, an invasive vector species in Florida, displaying the diagnostic gold patch on the anterior scutum [35]. (**B**) Screenshot of video posted to NASA Earth’s Instagram account (instagram.com/reel/CSzbNMyluzg, accessed on 30 June 2022). (**C**) Screenshots of press release materials from Mosquito Alert campaigns. (**D**) Screenshots of iNaturalist project page header (**left**) and geographic bounds (**right**), via campaign’s simplified URL mosquitoAI.org (accessed on 21 April 2022).

Our campaigns and apps were actively promoted by various means online and through social media. An initial multi-agency press release was published at the beginning of the campaign on 3 May 2021, followed by a news article by the University of South Florida (USF) on 20 August 2021 that featured the work of three student coauthors (C.M., D.D., and K.R.). Throughout Florida, outreach materials and campaign information involving all three apps were promoted via local television as well as the Twitter, Facebook, and Instagram accounts of USF, the Pasco and Miami-Dade County mosquito control districts, and the Florida Mosquito Control Association. We created “Wanted” posters for three of the target *Aedes* species (e.g., Figure 3A) that included an image, information, and labeled identifying markers for *Ae. aegypti* and *Ae. vittatus*. The *Ae. vittatus* image (not shown) was from a specimen previously trapped in Spain [57] and published shortly after the first detection of the species in Galicia via Mosquito Alert [43].

Mosquito Alert regularly carries out informational campaigns to encourage participation (Figure 3C). Until 2020, these were mostly focused on Spain, but with expansion into new countries, a model press release was created in October 2020 for use by teams in other countries. In 2021, a series of press campaigns generated participation in multiple countries in Europe through the course of the mosquito season (May–October). The Netherlands campaign was particularly successful, attracting large numbers of participants very quickly, as described further in the Results section, and placing pressure on the expert validation system. This has led the project to move toward a strategy that increasingly relies on AI, with a human-in-the-loop approach to validation.

The GLOBE Program periodically runs month-long data challenges to spur interest and excitement about the citizen science platform. From 25 July–25 August 2021, GLOBE ran a Mosquito Habitat Photo Challenge that was designed to encourage the practice of including coincident Land Cover observations as part of routine Mosquito Habitat Mapper observations. Other science objectives of the campaign were to increase the number of mosquito larva photos for use by our USF team in AI image recognition research, and to obtain land cover photos to support several project teams developing AI solutions to categorize land cover, funded by the NASA Goddard Space Flight Center’s Artificial Intelligence Center of Excellence (AI CoE). The Mosquito Habitat Photo Challenge was paired with a website (observer.globe.gov/do-globe-observer/challenges/mosquito-habitat-photo-challenge, accessed on 30 June 2022), a social media campaign, a series of informational webinars, and a Challenge Activity Tracker, where volunteers can log their participation in the event. In addition, a data dashboard was updated daily, presenting near-real-time progress during the challenge [45]. Materials were also created for communicating the utility of efforts and the instructions to citizen scientists (Figure 2; File S1). These instructions incorporated feedback from our initial AI training efforts. Namely, the need for users to take three sets of photos—full body, tail end, and head plus thorax—with all the hairs (i.e., setae) floating and all the photos in focus. An outreach video (Figure 3B) was also produced by one of the students (K.R.) as part of her honors thesis research and promoted globally through NASA Earth’s Instagram reels (1.6 million followers, 137,000 views, 3632 likes at time of publication).

Our iNaturalist campaign—covering the southern USA, the Caribbean, and Central and South America—began with a summer campaign (22 April–30 September 2021) and was continued for a full year to accommodate the summer months of the southern hemisphere (Figure 3D). During the summer campaign, the iNaturalist Journal feature was used to post four messages to the 120 members and other participants of the project, notifying and reminding them about the species and locations in the study, and encouraging them to submit additional observations. Focusing primarily within six Florida counties, we also directly contacted 264 iNaturalist users through the platform, asking them to join the project and contribute further observations and identifications of mosquitoes. This cohort included 119 users that we had identified as prolific “super users,” who were among the top 20 users in their county or region by either the number of mosquito observations or total observations.

### 2.5. Dashboard

To realize our current and future objectives, as well as to assess the value of citizen science reuse through a case study of mosquito monitoring, it was necessary to create a dashboard that displayed the location, imagery, category, and other relevant information from our various datasets in an interoperable, accessible, and visually appealing format. However, as discussed above, the incongruent nature of disparate data poses a large obstacle. Namely, a lot of great citizen science data have already been collected, but not in a way that facilitates interoperability with other projects. Therefore, all data needed to be harmonized according to the standard of an established platform, specifically that of the Global Earth Challenge initiative.

One goal of Global Earth Challenge, a citizen science platform for environmental research launched in recognition of the 50th anniversary of Earth Day, was to “Increase the amount of open and findable, accessible, interoperable, and reusable (FAIR) citizen science data to help researchers and policymakers understand how the environment is changing” (globalearthchallenge.earthday.org, accessed on 30 June 2022). Global Earth Challenge pursued this goal through two objectives: advancing work on the Open Geospatial Consortium (OGC)’s SensorThingsAPI data standard, and creating a new open data portal—the Citizen Science Cloud (cscloud-ec2020.opendata.arcgis.com, accessed on 30 June 2022).

The OGC is a standards development organization and global community committed to improving access to geospatial information (i.e., data with a location component). Under the OGC, a Citizen Science Domain Working Group (CS DWG; ogc.org/projects/groups/citizenscience, accessed on 30 June 2022) was chartered to support citizen science by providing a forum for communicating and demonstrating the benefits of using open standards and best practices. Members of the CS DWG conducted a number of interoperability exercises to understand how the OGC SensorThings API standard could be implemented to support the interoperable collection and/or exchange of citizen science data. These members included representatives from a range of citizen science projects and platforms, including COBWEB [58], SCENT (scent-project.eu, accessed on 30 June 2022), WeObserve (weobserve.eu, accessed on 30 June 2022), and most recently, Global Earth Challenge.

As documented elsewhere ([59]; ogc.org/standards/sensorthings, accessed on 30 June 2022), the current canonical version of the SensorThings API standard contains eight classes derived from the more general Observations and Measurements (O&M) standard. Some classes, such as “Location”, are designed primarily to support standardized data exchange. Other classes, such as “ObservedProperty”, are designed to support the standardized documentation of important information—such as the name, definition, and description of the subject of a citizen science research campaign—that may be traditionally considered information about a dataset, such as metadata. In addition to the eight classes typically encompassed in SensorThings API, the Global Earth Challenge implementation includes a ninth class, “OMProcess”, which includes documentation of information particularly relevant to citizen science, such as the data quality and intellectual property.

With all these principles and protocols in mind, the four data streams from Mosquito Alert, iNaturalist, Mosquito Habitat Mapper, and Land Cover were transformed to align with the Global Earth Challenge implementation of SensorThings API, and continuously pulled into the ArcGIS Online web application (Esri, Redlands, California, USA) as feature layers via an API. Data entries with invalid geometries were removed. The public duplicates of these feature layers were brought into the same map element in ArcGIS Online and then into the ArcGIS Dashboard creation tool. Pop-ups were configured using the Arcade expression language so that the images and accompanying information are displayed when a user clicks on an observation in the map window. ArcGIS Hub was used to enable public download of the geospatial data, and ArcGIS Experience Builder was used to provide users with shareable links as well as to optimize the mobile version of the dashboard to facilitate ease of access.

### 2.6. Artificial Intelligence

For identifying mosquito genera and species using a convolutional neural network (CNN), our Mosquito Alert team used a training dataset that combined adult mosquito images from Mosquito Alert (2014-21) and the Global Biodiversity Information Facility (GBIF; 2004-21, mostly from iNaturalist) to obtain a balanced redistribution across the targeted taxa. Before classification, we cropped the mosquitoes by selecting the best mosquito prediction from a ResNet50-backboned Faster R-CNN, and then resized this region to a 256 × 256-pixel image. Data augmentation was achieved by taking a random 224 × 224-pixel crop for each image and randomly flipping it so that we had a slightly different version of each image at each epoch. After cropping, we normalized the data using means and standard deviations from ImageNet. The CNN architecture was similar to that of [38] in employing an ImageNet pre-trained ResNet50 [60], except that we used a holdout validation with a randomly selected subset of 300 images for each class via pytorch.

In parallel, our USF team trained and tested separate artificial intelligence algorithms using mosquito images from all three apps herein, in conjunction with images of mosquitoes from traps and insectaries. For species prediction of adult mosquitoes, we used an Inception-ResNetV2 model based on that of [39]. A tenth class was added, *Ae. scapularis*, using 439 smartphone images taken from 10 specimens trapped and photographed by the Miami-Dade County Mosquito Control Section. Six specimens (303 images) were used for training, two specimens were used for validation (74 images), and two specimens were used for testing (62 images). We trained 100 more epochs and modified the following hyperparameters: optimizer (Adam [61]), learning rate (1 × 10^−5^), and batch size (8). For a given image, we also generated a class activation map (CAM; [39,62]), which is a heat map that illustrates the relative weighting of pixels used in the classification.

A second model was designed to automatically extract masks of pixels that correspond to specific anatomical components within an adult mosquito image. The purpose is to provide future utility for improving the species classification as well as the education and engagement of citizen scientists. Our framework was a mask region-based convolutional neural network (Mask R-CNN) [63]. To train this network, we used a ResNet-101 as a baseline model, based on that of [40]. We split the previous “thorax” mask into four masks (thorax, proboscis, head, and antenna) for a total of seven masks along with abdomen, wing, and leg. We then remasked 350 images from the previous dataset, and masked 300 of the aforementioned images from the 10 specimens of *Ae. scapularis*. The model was trained to 100 epochs with a 1 × 10^−3^ learning rate and then to 100 more epochs with a 1 × 10^−4^ learning rate. For additional testing of both models, we utilized iNaturalist images of *Ae. scapularis* from both before and after the start of the campaign, yielding a total of 44 images from 21 specimens.

## 3. Results

### 3.1. Campaigns

#### 3.1.1. Mosquito Alert

**Figure 4:**
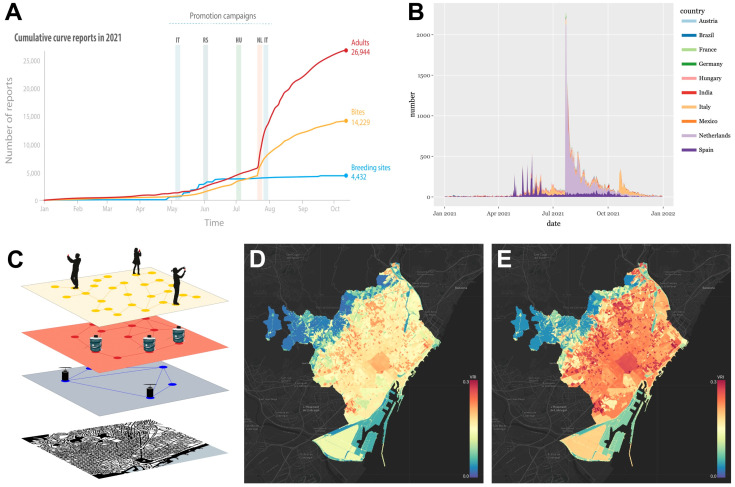
Mosquito Alert reports and risk model. (**A**) Reporting dynamics and sequence of key outreach events in the EU 2021 Mosquito Alert Campaign for Italy (IT), Serbia (RS), Hungary (HU), the Netherlands (NL), and Italy (IT). The success of the Netherlands outreach campaign raised the cumulative number of reports from 5000 to more than 25,000 reports, most of them occurring in a couple of weeks. (**B**) Time series area plot showing the total number of reports over time from the 10 countries with the highest number of reports in 2021. (**C**) Schematic of data layers used to derive *Aedes albopictus* risk maps. Maps are based on a set of ensemble models that combine Mosquito Alert citizen science data (**top**) with data from traditional adult mosquito traps and AI-driven smart traps—and also include data on weather, land cover, and sociodemographic characteristics (not shown)—to calculate the vector risk index (VRI) and project it onto a street map layer (**bottom**). The VRI is a measure of relative risk of human contact with *Aedes albopictus*, shown here for Barcelona, Spain, on 8 (**D**) and 15 (**E**) September 2021. The live version of this map is available at mosquito-alert.github.io/MosquitoAlertBCN/, accessed on 30 June 2022.

In 2021, Mosquito Alert received 51,560 reports from 18,680 participants in 152 countries. Of these, 31,129 were reports of adult mosquitoes, 4547 were reports of mosquito breeding habitats, and 15,884 were reports of mosquito bites. Most of the reports came from the Netherlands, where the information campaign generated a very rapid surge in participation at the end of July 2021. In total, Mosquito Alert received 22,154 reports from the Netherlands in 2021. These reports came from 8379 participants and were almost entirely of mosquito adults (16,554) and bites (5394). Figure 4A,B show a time series of the number of reports received in 2021 from each of the 10 countries with the highest number of reports that year.

Data from the app continues to contribute knowledge about the distribution of autochthonous mosquito species. The Mosquito Alert platform also generates web maps with real-time and forecasted risks of vector (*Ae. albopictus*) exposure for the city of Barcelona (Figure 4C–E; mosquito-alert.github.io/MosquitoAlertBCN/, accessed on 30 June 2022) and across Spain (mosquito-alert.github.io/MosquitoAlertES/, accessed on 30 June 2022) by combining live citizen science reports with data on weather, land cover, and sociodemographic characteristics. The Barcelona risk maps combine the citizen science estimates with estimates made from a network of both traditional mosquito traps and AI-driven smart traps, which automatically identify mosquitoes as they fly in. Combining these data streams has proven valuable because of their complementarity, and the approach is now being scaled up to cover larger geographic areas.

#### 3.1.2. GLOBE Observer

Between 2017–2021, citizen scientists worldwide submitted 32,845 Mosquito Habitat Mapper observations and 17,334 Land Cover observations. These data are openly available for visualization and download at globe.gov/globe-data (accessed on 30 June 2022). A summary of Mosquito Habitat Mapper data from 2017–2020 is reported in [17] (see also Low et al., 2022 [45]). In 2021, the month-long summer photo challenge stimulated the submission of 539 larval photos (Figure 2), 1360 habitat photos, and 5125 land cover photos. This included an unprecedented 127 records with coincident observations from both Mosquito Habitat Mapper and Land Cover tools. All participants were able to download a customized certificate of participation in the challenge and obtain a digital badge.

#### 3.1.3. iNaturalist

During our summer campaign, the first two observations of *Ae. vittatus* on the iNaturalist platform were submitted. Four observations of *Ae. scapularis* were also submitted during this time; all six observations are Research Grade. During the remainder of our annual campaign, 20 additional Research Grade observations of *Ae. scapularis* were submitted. Notably, this included the first iNaturalist observations of the species in the USA (n = 4; Hidalgo County, Texas). To date, that county includes the only iNaturalist observations of *Ae. scapularis* in the USA, comprising a total of five specimens observed by four citizen scientists within ~40 km. These discoveries of the invasive vector *Ae. scapularis* were shared with the Hidalgo County Health and Human Services Department responsible for mosquito surveillance and control. It should be noted that an external super user recruited to our project was responsible for the initial species-level identifications of the first three global *Ae. vittatus* and the first three USA *Ae. scapularis* specimens on the iNaturalist platform.

Within the four Florida counties of Hillsborough, Pasco, Polk, and Pinellas during the summer campaign period, there were 239 iNaturalist observations of mosquitoes, which included 23 Research Grade observations of *Ae. aegypti* representing six unique locations. For validation of the trap-based habitat model, these six locations were combined with the four other Research Grade *Ae. aegypti* observations/locations in this region during the summer months (April–September) from 2017–2021. No Mosquito Alert observations for *Ae. aegypti* were present in this region during these periods. While the modeling and validation results will be published in a future manuscript, here we report on the observer bias of these citizen science data, given that such observations will generally occur in places where there are more people. The census tract-level population density for 9/10 of the locations was above that of the respective county by an average factor of 3.3 (range: 0.4–11.2). We should also note that the outlier was one of the two locations that we had contributed (D.D.).

### 3.2. Dashboard

**Figure 5:**
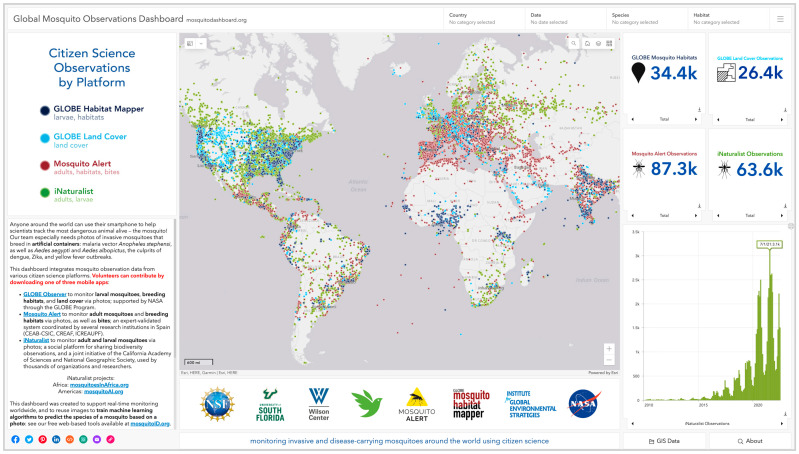
Screenshot of the Global Mosquito Observations Dashboard (mosquitodashboard.org, accessed on 30 June 2022). This interactive dashboard combines various types of observations from our four data streams into an interoperable visualization. Each color-coded dot represents a citizen scientist’s observation and can be clicked to access the associated photos and data. Full, filtered, summarized, and geospatial datasets can be downloaded, thus enabling data reuse.

Our cross-platform integration efforts culminated in the Global Mosquito Observations Dashboard (GMOD; Figure 5), openly accessible at mosquitodashboard.org (accessed on 30 June 2022). Text and links provide visitors with information about our dashboard, apps and organizations, and ongoing campaigns and projects. A legend lists the four data streams and various types of observations present on the map, which presents a global visualization of the continuously updating, interoperable citizen science mosquito data. Observations can be selected across multiple platforms simultaneously by using one or more filters: country, date range, species and genus, and breeding habitat. For calculating observation counts per platform within custom geographic selections, lasso and shape tools are available. Users can navigate within the map by typing in an address or place, as well as toggle the visibility of each data layer, customize the base map layer, and adjust and maximize panel frames. Indicator panels display total and category counts, and dynamic graphs that respect the filters visualize important trends within each dataset, such as growth in platform usage.

Clicking a color-coded dot on the map generates a pop-up window that displays data from that observation, along with the associated original photo(s)—mosquito adult, larva, habitat, or land cover—which can be opened in a new browser tab or downloaded. To facilitate data reuse, the full and filtered datasets as well as monthly summaries can be downloaded as CSV files directly from the dashboard. An ArcGIS Hub interface provides for the exporting of feature services in a variety of geospatial formats (i.e., CSV, KML, shapefile, GeoJSON, and file geodatabase). Users can seamlessly share the dashboard via links to social media platforms, email, and embed or QR codes.

### 3.3. Artificial Intelligence

**Figure 6:**
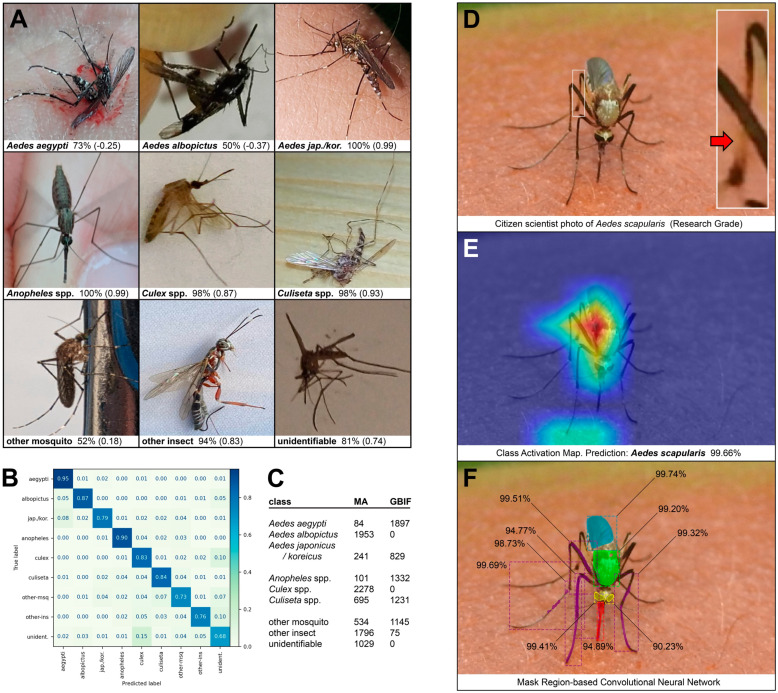
Artificial intelligence (AI) analyses of citizen science photos. (**A**) Mosquito Alert (MA) submissions correctly classified by AI, as verified by entomologists. The percentage is the model’s prediction confidence. The number in parentheses is the difference between the prediction confidence and the threshold of acceptance selected from the receiver operating characteristic curve (a value between −1 and 1, where 0 means that the prediction confidence is on the threshold). (**B**) Confusion matrix of classification accuracies from the validation set. (**C**) Number of images from the MA and Global Biodiversity Information Facility (GBIF) databases that were included in the training set. (**D**) Photo of *Aedes scapularis* submitted to iNaturalist during our summer campaign, modified here with a white box and inset. In addition to the diagnostic gold patch on the anterior scutum, the diagnostic pale patch on the anterior hind tibia is also visible (inset). (**E**) Class activation map, illustrating the relative weighting of pixels used in the species classification; warmer colors correspond to higher weights. Visualization represents the feature map from the last convolutional layer, overlayed at 50% opacity onto a grayscale version of the original photo. We applied a threshold of 110 to the feature map to show only those pixels that have contributed most to the final output. The percentage is the model’s prediction confidence. (**F**) Anatomical regions automatically identified using a mask region-based convolutional neural network approach [40]. Results yielded localization (dashed bounding box) and pixel-wise segmentation of each region: proboscis (red), head (yellow), thorax (green), wing (blue), and leg (purple). Prediction confidence (percentage) for each region was manually overlaid onto the results image.

Preliminary results are shown below from our Mosquito Alert (Figure 6A–C) and USF (Figure 6D–F) teams. The former panels include representative test images of dead or alive specimens, a confusion matrix of classification results, and a table enumerating the training images used for the nine classes. Results demonstrate high classification accuracy, with *Ae. aegypti* exhibiting the highest (95%). Figure 6D shows one of the Research Grade images of *Ae. scapularis* submitted to iNaturalist during the summer campaign (11 August 2021; Honduras) and used in the testing dataset. Visible are the two most salient diagnostic features of this species [35]: the gold patch on the anterior surface of the scutum and the pale patch on the anterior surface of the hind tibia (arrow in inset). Notably, these two regions were highlighted by the CAM (Figure 6E), signifying the relative importance of these pixels in the model’s species classification. The model correctly classified this image as *Ae. scapularis* with 99.66% confidence. Validation accuracy was 90.54% for the *Ae. scapularis* class and 92.40% for the 10-class model overall (cf. 80% for the previous 9-class model; [39]). Results from the Mask R-CNN model demonstrate that the anatomical regions were accurately identified and with high confidence (Figure 6F). Mean confidence for this image was 97.55%. At 30% intersection over union (IoU), the mean average precision (mAP) of the validation dataset (200 images) was 39.47%. Results from the AI research using Mosquito Habitat Mapper larval images will be published in a future manuscript.

## 4. Discussion

Citizen science offers a globally scalable, cost-effective solution for the real-time monitoring of mosquito populations of public health concern. To help actualize this vision, we achieved three objectives: (I) integrated different observational data streams into an interoperable dashboard, with utility for researchers, mosquito control personnel, and policymakers; (II) leveraged citizen science platforms for vector modeling and the successful detection of targeted invasive species; and (III) reused images for training and testing a variety of novel AI solutions. These results demonstrate the value and potential of our Global Mosquito Alert Consortium paradigm of bringing together international projects to promote data sharing, interoperability, and reuse.

One advantage of using citizen science to answer complex research questions is the community’s decentralized nature—allowing a diversity of projects to reach success in all corners of the world. Projects can be local or global by design, which presents an opportunity for investigating more geographically ambitious and cross-disciplinary topics than a single project could address alone. Yet such decentralization is also a fundamental hindrance unless canonical protocols and practices are adopted, ideally incorporated into the project design and process from the inception.

Many researchers have explored how different characterizations of the citizen science research process influence whether and to what degree citizen science data have sufficient quality to be fit for an intended use. For example, Stevenson and colleagues recently published a taxonomy of citizen science data types associated with different data quality requirements and methods [64]. Shirk et al. emphasized a framework for deliberate design to enhance the quality of public participation and scientific research outcomes [65]. Others have explored how various data collection, curation, and management practices more broadly inform “the state of the data in citizen science” [66]. Such work is helpful not only for understanding whether the data associated with a particular citizen science project can achieve an initial set of goals or outcomes, but also for elucidating the conditions that enable (or inhibit) data reuse.

A few researchers have directly focused on the question of how to maximize data reuse and impact. Williams and colleagues identified four key factors to consider when planning a citizen science project: *data contextualization*, or “communicating the context in which a particular dataset has been created” to allow “users to evaluate its possible reuse”; *data interoperability*, or “enabling seamless reuse of resources [...] across different systems”; ensuring and communicating *data quality* and reliability; and, ultimately, promoting ongoing *data reuse* through clarifying data ownership and securing future accessibility ([67], p.322). These researchers concluded, “To maximise the impact and reusability of citizen science data, citizen science projects should therefore adopt standards for web services or data encodings and, where possible, adapt previously collected observations to these standards.” ([67], p. 335). Other complementary studies (e.g., [68]) emphasize that standards should encompass both data structure and (meta-)data documentation, and that reuse requires making data both open and FAIR [69]. Once such a standard is implemented, additional metadata attached to each data point will serve as an advantage rather than an impediment. Ultimately, more work is needed to build on these efforts to understand the potential and use cases associated with data reuse, the specific steps that can be taken to enable reuse, and potential barriers that may arise.

For us, data interoperability was both a challenge and an opportunity. Transforming disparate established datasets to match a new data standard was an enormous undertaking that posed the largest barrier to getting the dashboard fully functional, with all four data streams being displayed and actively updating in real time. These difficulties only strengthen the argument for a stronger push to standardize the data collection of existing and emerging research-grade citizen science projects. It should be noted that the dashboard presented is only the first step in our research group’s collective goal for the integrated platform. Currently, the visualization provides the first comprehensive, continually updating representation of mosquito monitoring data from multiple global citizen science projects (Figure 5). To facilitate opportunities for reuse, these harmonized data are open and FAIR, and hosted within an interactive interface that serves as a free practical tool for the synthesis, filtering, and exporting of data.

By aggregating various types and modalities of data, this dashboard diversifies the portfolio of approaches and combines the strengths of each citizen science project. For example, Mosquito Habitat Mapper and Mosquito Alert are mosquito-specific, whereas iNaturalist is more generalized but with a larger userbase and crowdsourced identifications. Together, voluminous data are captured from across the mosquito life cycle: potential and actual breeding habitats, larvae, adults, and human interactions (bites). Integrating these data can enable future analyses that elucidate connections between certain environments and the presence of certain mosquitoes. One example of this cross-platform synergy is the coincident Mosquito Habitat Mapper and Land Cover observations. These will help meet the need for fine-grained data describing the spatial relationship of breeding habitat locations and the land cover classes identified in satellite products (cf. [70]). Specifically, these data provide high-resolution land cover features immediately surrounding larvae and water sources, enable the identification of microhabitat and microclimate features undetectable in Landsat (30 m) or Sentinel (10 m) image products [71], and have the potential to improve the predictive power of mosquito-borne disease risk models, which frequently employ land cover data obtained from satellites (see systematic review by [72]).

While investigations into the degree to which citizen science can aid in the construction of risk and habitat models are at an early stage, models that have already been constructed from trap data may benefit from validation by citizen science data—especially when combining datasets to account for certain spatial biases. Of course, citizen science data has its own spatial biases, particularly the association with population density. Nonetheless, citizen science can play an important role in validating models because such data are not subject to the same placement biases as trap data may be. In these cases, it may be prudent to factor both citizen science data and trap data into a holistic validation, with the trap data validating abundance and the citizen science data validating the probability of presence.

In conjunction with trap-based monitoring, particularly in cases in which the traps are limited to a known boundary of the species’ habitat, citizen scientists can make observations outside of these bounds to provide real-time updates of potential further invasion. Traditional mosquito monitoring requires that traps are placed and maintained in various target locations. Because surveillance programs are carried out at different levels of jurisdiction, mosquito surveillance may not be coordinated or standardized on a county-to-county or state-to-state level, which may result in different traps being used between different entities, different target species being observed, or needless overlap in surveillance. The deployment and maintenance of such traps also require funding, the stability of which is not guaranteed from one jurisdiction to the next. However, citizen science platforms can exhibit a greater degree of spatial flexibility in detecting the location of species of concern, and can then inform the aforementioned entities on where additional trap-based monitoring or vector control measures may be necessary, as is the case in Spain [14]. Thus, programs may benefit from a positive cycle of both active and passive monitoring, especially given their complementary nature [25].

Our dashboard enables such utility of passive data, and the real-time mapping of mosquito presence has broad implications for early warning systems that focus on vector management to control or prepare for disease spread. Integrating data from multiple projects ameliorates the otherwise limited spatial coverage and lifespan of projects that have a shorter-term focus (e.g., the Zika epidemic of 2015–2016) and/or do not have sustained funding. Combining the outputs of different citizen science platforms can also hedge against disruptions or fill gaps in usage where they may occur due to country-specific projects. For example, many citizen scientists in Spain use Mosquito Alert, offering good surveillance of targeted species across the country. Far fewer citizen scientists use Mosquito Alert in Portugal and France, but there is a large iNaturalist userbase in both countries, effectively expanding the possible extent of cohesive analysis when complementary data from both platforms are brought together.

For such purposes, the dashboard can visualize the growth in the number of citizen science observations, identify which platforms are more popular in different regions, and determine which regions have multiple platforms that are well-represented–all of which can be useful for informing future campaigns, monitoring activities, and scientific analysis. Such an integrated visualization also helps to identify geographic regions with little or no coverage by any of the platforms. Notably, there is a lack of mosquito observations in much of Africa (Figure 5). This is especially critical in countries such as Ethiopia, given the recent invasion of the urban malaria vector *An. stephensi* and the profound risk that this represents [73]. This problem species, already well-established in Asia, is a focus of our team’s current and future work (e.g., mosquitoesinafrica.org, accessed on 30 June 2022), especially with respect to larval surveillance and AI tools (below).

Combining such geographically diverse platforms is particularly important for mosquito species with current or future ranges that span those of different user communities. For example, given the initial absence of iNaturalist observations of *Ae. vittatus*, we leveraged existing images and specimens (*n* = 27 and 4) from the Mosquito Alert platform for AI training. While not yielding any additional *Ae. vittatus*, we also reviewed 51 images from 29 specimens of unidentified *Aedes* spp. in the Mosquito Alert system. Subsequently, the first iNaturalist observations of *Ae. vittatus* were made during our targeted summer campaign, contributing a total of six images from two specimens. Together, the iNaturalist and Mosquito Alert images of *Ae. vittatus* are being used in ongoing training for the next iteration of the USF models. These efforts demonstrate the beneficial network effect—whereby an image generated on one platform is useful for another—as well as the utility of international collaboration, given that this vector species is invasive to the New World but present in the Old World (and thus more abundant to image for training).

Globally, the highest peak in monthly mosquito observations that has occurred to date on the Mosquito Alert and iNaturalist platforms happened during July of our summer campaigns (Figure 4A and Figure 5; 13,400 and 3100 respectively). Successful results from our annual campaign included 24 new iNaturalist observations of *Ae. scapularis*, which exceeded the number of observations for that species over the previous six years combined (n = 19, all Research Grade). These two dozen submissions included the platform’s first four observations of the species in the USA—within a single Texas county—and provided actionable surveillance data. No *Ae. scapularis* observations were made in the two Florida counties in which the species has recently invaded (Miami-Dade, Broward) [35]. However, within these two counties during our summer iNaturalist campaign, there were 122 mosquito observations (42 Research Grade) from at least 17 species. Our promotional strategy there and elsewhere in Florida was two-pronged: top-down social media campaigns from a variety of organizations, coupled with bottom-up direct contact with iNaturalist users and indirect contact through the project’s Journal feature. Hopefully, these efforts also served to prime citizen scientists for the future detection of *Ae.* *scapularis* and other mosquitoes of concern.

Overall, communicating the scientific utility of observations through such direct engagement seemed to motivate the super users and other participants (pers. obs. R.M.C.). Indeed, when volunteers know that their data is meaningful and will be used in research, they are more likely to continue making citizen science observations [74]. Toward that end, and to improve the usefulness of citizen science data by the scientific community, the GLOBE Observer app has developed a geofencing tool that allows scientists to specify map polygons where they need data, and alerts citizen scientists that they are located in or near an area where a project has requested data collection. The development of such in-app tools to improve the ease of use by citizen scientists and the data access by research scientists creates critical connections between the two groups. Future engagement will involve encouraging users to recruit new users to the platform, not just to the particular project, as well as leveraging local non-governmental organizations and communities of naturalists.

One substantial limitation throughout the course of our project was the ongoing COVID-19 pandemic. Logistically, this global emergency affected our citizen scientist efforts in a variety of ways, including having to abandon plans for “bioblitz” [75] community events targeting specific mosquitoes and areas of interest. The effects of the pandemic on citizen science participation itself were undoubtedly complex and context dependent as well. For iNaturalist observations made in 2020, participants overall did not travel as much, but they continued making new observations at a similar proportion to that before the pandemic (rather than shifting most activity to posting observations from years past) [76]. During our 2021 summer campaign in the Tampa Bay area, the pandemic may have contributed to the paucity of *Ae. aegypti* observations (albeit still representing a relative increase compared to prior years), the key limitation in the habitat model validation. Conversely, Mosquito Alert observations increased over 2020–2021 (Figure 4), although this was due in part to a dramatic increase in the Netherlands after the promotion of the app over national television and radio by politicians there. Mosquito Habitat Mapper observations decreased compared to baseline pre-COVID data, which can be attributed in part to health messaging by the GLOBE Observer team: data collection was actively discouraged during the first year of the pandemic to reduce opportunities for COVID contagion.

The potential downstream effects of this global pandemic on various mosquito populations and mosquito-borne diseases require further study as well; for example, changes in the abundance and types of artificial containers serving as mosquito breeding habitats during quarantine, the shift of human activity indoors, and tradeoffs in resources and attention directed toward pandemic efforts. Additionally, the World Health Organization estimates that service disruptions during the COVID-19 pandemic resulted in an estimated 47,000 additional deaths from malaria in 2020 [12].

Looking forward, one of the most promising directions for our research is the reciprocal leveraging of the vast wealth of citizen science image data and AI techniques for identification. For example, cross-platform integration of digital data from various citizen science programs was important for creating sufficient and balanced training image datasets. Application of the resulting algorithms to citizen science images was shown to provide accurate and useful techniques for identifying species and anatomy, even among smashed specimens. With respect to the taxonomic identification of mosquito observations, future development and deployment may improve the reliability of citizen scientist submissions and decrease reliance on expert validation. Additionally, we are making our AI tools openly available as an early warning system for the surveillance and control of invasive and vector mosquitoes, and in particular *An. stephensi* in larval and adult stages: mosquitoid.org (beta, accessed on 30 June 2022).

As shown in Figure 6E, the CAM highlighted the mosquito—and pixels of both the diagnostic scutum and hind tibia in particular—as most important in the correct classification of our target species, *Ae. scapularis*. Such a heat map enables “explainable AI”, by providing visual information of the algorithm’s computational result as well as critical validation that the relevant pixels are being used. The potential exists for such AI techniques to yield new anatomical insights, perhaps even informing future identification keys for mosquito species. We were also able to accurately extract the core anatomical components of mosquitoes (Figure 6F). The next step will be to design AI algorithms geared toward “anatomically informed deep learning”, with region-specific models that we anticipate will improve the accuracy of species classification. In the future, if anatomies were extracted and visualized in real-time, this feedback could pique engagement as well as educate users on mosquito anatomy (e.g., diagnostic traits) and perhaps even provide utility for training mosquito control personnel.

In addition to species and anatomical identification, AI can also provide utility for image validation upstream in the process. In 2022, the GLOBE Observer Mosquito Habitat Mapper system will migrate to a photo validation process driven by AI. When this process is in place, any photo that is flagged by the AI will be reviewed manually prior to being rejected or approved. Similarly, Mosquito Alert will add AI to its validation system, to complement and streamline the work of the experts who currently validate all adult mosquito reports. The hope is that this will make the system more scalable while maintaining data accuracy at adequate levels for vector management.

## 5. Conclusions

Based on our experiences herein, we offer five recommendations for the planning and execution of similar citizen science efforts: (1) create visual materials for outreach and instruction (the “what”); (2) communicate to citizen scientists the research utility of the project data (the “why”); (3) contact super users directly to request their participation and recruitment of others (the “who”); (4) evaluate previous and ongoing platform usage within the project’s spatial bounds (the “when” and “where”); and (5) standardize data and metadata collection using established structures and protocols to best enable seamless cross-platform integration and reuse in the future (the “how”).

In these ways, existing sociotechnologic infrastructure can best be leveraged, by harnessing both online and on-the-ground communities of citizen scientists equipped with georeferenced computers in their pockets (i.e., smartphones). The accuracy and acuity of such integrations will only improve with continued technological advances in computer vision and the “eyes” of smartphone cameras. For example, the advent of built-in macro capability for such mobile devices will eliminate the need for a clip-on lens, and thus remove a primary bottleneck. The implications such developments have for public health measures and preparedness are bolstered by AI’s ability to identify mosquito species—especially considering that relatively few species can transmit diseases that warrant monitoring.

As the climate changes and the environment changes with it, fluctuations in biodiversity can be documented through global citizen science efforts. Vector tracking and management contribute to our evolving understanding of not only the changing world but also what that means for health outcomes, especially from a One Health perspective. This type of cross-disciplinary approach to public health is becoming the new standard for the understanding of disease, especially in light of the COVID-19 pandemic and its probable zoonotic origin. Ultimately, coordinated efforts can realize new synergies from international collaborations across various institutions, allowing fully harmonized datasets to resonate across the world in powerful and unforeseen ways for researchers, mosquito control personnel, and policymakers. Together, citizen science and artificial intelligence enable a next-generation surveillance framework that can serve as a global monitoring and early warning system for years to come.

## Data Availability

The harmonized data from this study are openly accessible and available for download from our online dashboard at mosquitodashboard.org (accessed on 30 June 2022). Reuse of this data falls under the respective Creative Commons licenses of the original platforms and/or individual observations. Mosquito Alert data are available from the Mosquito Alert Data Portal at mosquitoalert.com/en/access-to-mosquito-alert-data-portal (accessed on 30 June 2022), with the images available here mosquito-alert.github.io/metadata_public_portal/meta_ipynb/tigapics.html (accessed on 30 June 2022), all licensed under Creative Commons CC0 1.0 Universal (CC0-1.0). GLOBE Observer data analyzed in this project are publicly available at globe.gov/globe-data (accessed on 30 June 2022). The Python code to read, analyze, and visualize GLOBE data for this article as well as the analyzed datasets are available on github.com/IGES-Geospatial (accessed on 30 June 2022). Dashboard access to Mosquito Habitat Mapper and Land Cover data is available at geospatial.strategies.org (accessed on 30 June 2022). To best enable reuse with attribution, all data products from the Earth System Exploration Portal are licensed under Creative Commons Attribution 2.0 Generic (CC BY 2.0). iNaturalist data is available at inaturalist.org (accessed on 30 June 2022) as well as the Global Biodiversity Information Facility [53].

## Supplementary Materials

The following are available online at https://www.mdpi.com/article/10.3390/insects13080675/s1, File S1: Mosquito Habitat Mapper photo tips handout.
