# Supplementary material for: Integrating Global Citizen Science Platforms to Enable Next-Generation Surveillance of Invasive and Vector Mosquitoes
Source: Insects. 2022 Jul 27;13(8):675. doi: 10.3390/insects13080675 (PMC9409379; doi:10.3390/insects13080675)
Supplement: Supplementary file 1 [file insects-13-00675-s001.zip › insects-1687741-supplementary.pdf]

Mosquito Habitat Photo Challenge

July 25 to August 25, 2021

GLOBE Observer

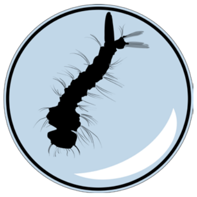

Help Improve Mosquito Disease Prediction Maps

Photos submitted during this challenge will be used to create automated classification programs that can identify mosquito larvae and the environments they prefer. Your photos of mosquito larvae, the habitats where you find them, and the land cover around the area are critical pieces of a complex environmental story.

TIPS

Taking Mosquito Larva Photos

Turn page over to find six PRO TIPS on taking high-quality mosquito larvae photographs

Step	Looks Like This	Tips	Why it Matters
**1. Start with the Mosquito Habitat Mapper tool.** Invite family and friends to join the **Mosquito Habitat Photo Challenge**.	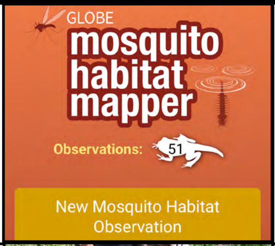	**Download the GLOBE Observer app**, which you will use to upload your information. ***observer.globe.gov***	Using a **smart device** allows you to take photographs of mosquito larvae, their habitats and land cover, and **upload these observations for analysis**.
**2. Find a Habitat.** Mosquitoes like stagnant, still, non-flowing water. It could be either natural or an artificial container.	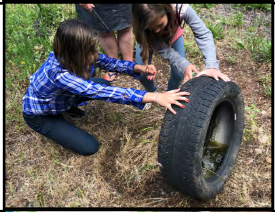	Places to look include **tires**, **birdbaths**, **trash**, and any type of **open water storage containers**. Also look for places where water may pool up undisturbed.	Female mosquitoes look for **water sources** to lay their eggs. When the eggs hatch, larvae emerge. Any place water has collected could become a mosquito habitat.
**3. Sample the water.** Scoop water sample into a cup or use a turkey baster. Place any larvae on a white background like a paper plate.	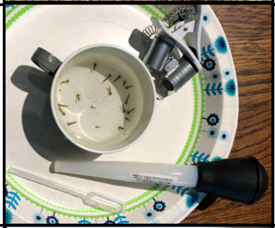	**Avoid casting shadows** over the water (otherwise larvae may think you are a predator and dive below the surface). Skim the water's surface with a cup or turkey baster.	Mosquito larvae are small and usually actively wriggling around. **Select just one or two mosquito larvae to photograph**.
**4. Photograph larvae.** Take at least 6 photos ***(see photography details on next page).***	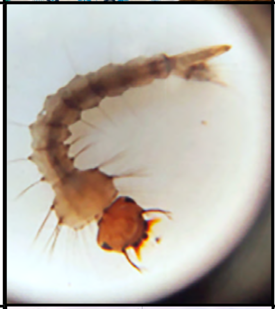	**Take six larva photos:** 2 of the full body and hairs<br>2 of just the head and hairs<br>2 of the tail and hairs<br>***See larva photography details on the next page***	**Scientists will use your photos** to create a classification program that identifies the genus of mosquitoes by unique traits of the larva's head, siphon (breathing tube), and position and numbers of hairs. It is important to **take clear, in-focus photos** using a clip-on magnifier.
**5. Take land cover observation photos.** Do this every time you find a larval habitat site.	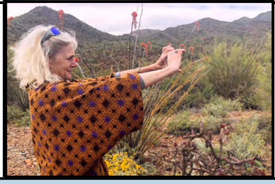	**Launch the Land Cover tool.** Take 6 photos *(North, South, East, West, up and down).* For this challenge, classification is not necessary.	Scientists are interested in the land cover (water area) where larvae are found, so they can **automate image classification** using Artificial Intelligence (AI).

Remember to upload your observations using the GLOBE Observer app • observer.globe.gov

Learn More

observer.globe.gov/mosquito-challenge

nesec NASA Earth Science Education Collaborative

NASA Partner

Using a Clip-on Magnifier*

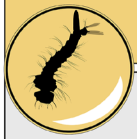

Picking larva: Mosquito larvae are small (growing up to 1/2 inch). Pick the largest larva in your sample and use a clip-on magnifier (capable of at least 60-100x) with a smartphone to photograph.

✱ **Note:** If your magnifier is different from the one shown in this graphic, follow its directions for use.

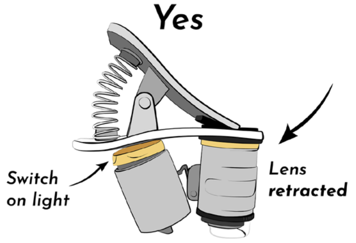

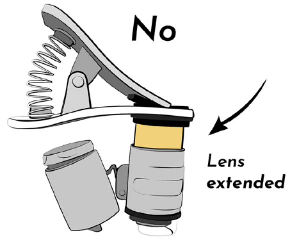

1 *Remove phone case and clip on the lens (**retracted** position).*

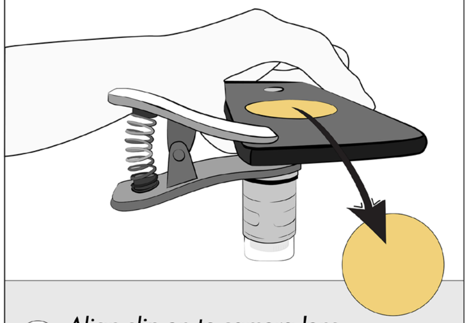

2 *Align clip-on to camera lens to find a **perfect circle view** (you won't see larva specimen yet).*

3 *Keeping perfect circle, **lower phone** until **clear plastic collar rests directly on your plate's surface.***

Ready, set … snap your photo!

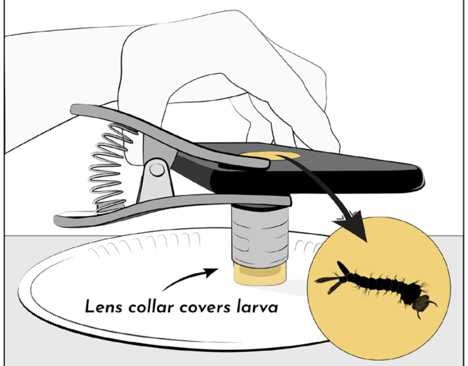

TIPS Six Pro Tips for Photographing Your Larva Specimen

Remember to upload your observations!

1. Use a **pipette** (or dropper, straw, spoon) to obtain one larva. Pour small water sample on white plate.

2. **Isolate one larva. Make sure it is suspended and completely covered in a drop of water** so its hairs float and extend naturally. Too little water will make the hairs stick to the sides of the larva and may also cause it to dry out. A drop of hand sanitizer will slow larva activity.

3. **Phone Focus Tips**

Clip macro lens over camera lens, lining them up to see a **perfect circle** of light on phone screen.

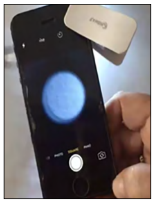

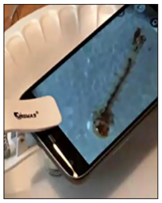

Digitally zoom on the phone (e.g., pinch-drag on screen). Tap screen to ***refocus***. Repeat zoom (pinch-drag) process for your six larva photos ***(see steps 4-6 below).***

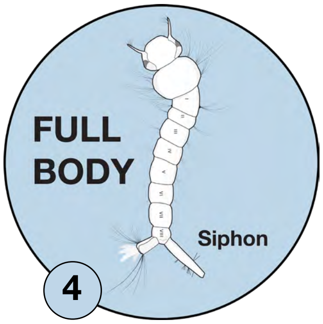

Take **TWO PHOTOS** of larva's **FULL BODY**, including **all HAIRS ("setae") - *in focus.***

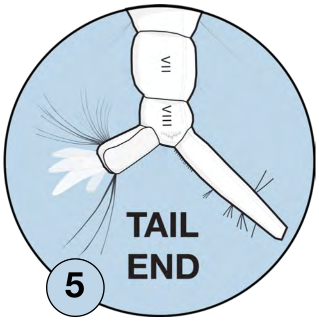

Tap screen again to ***refocus***. Take **TWO PHOTOS of the TAIL END+HAIRS - *in focus.***

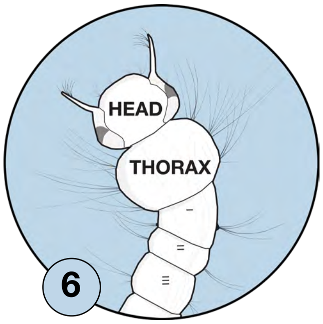

Move lens to the head. Tap screen to ***refocus***. Take **TWO PHOTOS of HEAD+HAIRS - *in focus.***

Learn More

observer.globe.gov/mosquito-challenge

nesec NASA Earth Science Education Collaborative

NASA Partner
